# Relevance of dietary protein concentration and quality as risk factors for the formation of calcium oxalate stones in cats

**DOI:** 10.1017/jns.2014.13

**Published:** 2014-11-07

**Authors:** Nadine Paßlack, Hannes Burmeier, Thomas Brenten, Konrad Neumann, Jürgen Zentek

**Affiliations:** 1 Institute of Animal Nutrition, Department of Veterinary Medicine, Freie Universität Berlin, Königin-Luise-Str. 49, 14195 Berlin, Germany; 2 Mars GmbH, Eitzer Straße 215, 27283 Verden, Germany; 3 Institute of Biometry and Clinical Epidemiology, Charité – Universitätsmedizin Berlin, Hindenburgdamm 30, 12203 Berlin, Germany

**Keywords:** Cats, Dietary protein, Protein quality, Renal calcium and oxalate excretion, Urinary pH, BW, body weight, CaOx, calcium oxalate, CP, crude protien, HQ 35 %, high protein quality diet with 35 % CP content, HQ 44 %, high protein quality diet with 44 % CP content, HQ 57 %, high protein quality diet with 57 % CP content, LQ 55 %, low protein quality diet with 55 % CP content, Ox, oxalate, RSS, relative supersaturation

## Abstract

The role of dietary protein for the development of feline calcium oxalate (CaOx) uroliths has not been conclusively clarified. The present study evaluated the effects of a varying dietary protein concentration and quality on critical indices for the formation of CaOx uroliths. Three diets with a high protein quality (10–11 % greaves meal/diet) and a varying crude protein (CP) concentration (35, 44 and 57 % in DM) were compared. Additionally, the 57 % CP diet was compared with a fourth diet that had a similar CP concentration (55 % in DM), but a lower protein quality (34 % greaves meal/diet). The Ca and oxalate (Ox) concentrations were similar in all diets. A group of eight cats received the same diet at the same time. Each feeding period was divided into a 21 d adaptation period and a 7 d sampling period to collect urine. There were increases in urinary volume, urinary Ca concentrations, renal Ca and Ox excretion and urinary relative supersaturation (RSS) with CaOx with increasing dietary protein concentrations. Urinary pH ranged between 6·34 and 6·66 among all groups, with no unidirectional effect of dietary protein. Lower renal Ca excretion was observed when feeding the diet with the lower protein quality, however, the underlying mechanism needs further evaluation. In conclusion, although the observed higher urinary volume is beneficial, the increase in urinary Ca concentrations, renal Ca and Ox excretion and urinary RSS CaOx associated with a high-protein diet may be critical for the development of CaOx uroliths in cats.

The role of dietary protein for the development of calcium oxalate (CaOx) uroliths in cats has not been conclusively clarified. In human subjects, an increased renal oxalate (Ox) excretion has been found to be associated with a higher protein intake^(^^1^^)^; however, contrary results have been observed in cats^(^^2^^,^^3^^)^. Up to now, the reasons for these contradicting effects in human subjects and cats remain unclear. Zentek & Schulz^(^^2^^)^ hypothesised that the observed decrease in urinary Ox concentrations when feeding the high-protein (but low-fat) diets compared with the low-protein (but high-fat) diets to the cats could possibly not result from the protein, but from the fat concentration of the diets. Dijcker *et al.*^(^^3^^)^ also observed the highest urinary Ox concentrations in cats when feeding a high-fat diet compared with a high-protein and high-carbohydrate diet. Thus, one explanation for the contradicting effects of dietary protein on the urinary Ox concentration and excretion reported in human subjects and cats could be the variation in the fat concentrations of the experimental diets. This hypothesis is supported by findings in human subjects, where a high dietary fat intake has been identified to increase renal Ox excretion^(^^4^^,^^5^^)^.

Besides urinary Ox concentration and excretion, further factors have been discussed as potential risk factors for the development of CaOx uroliths in cats, including urinary volume, urinary pH and urinary Ca concentration and excretion. In general, a greater urinary volume lowers the total concentrations of Ca and Ox in the urine^(^^6^^)^, assuming that the renal excretion of Ca and Ox remains unchanged. Since increasing dietary protein concentrations were associated with increased water intake and a higher urinary volume in adult cats^(^^7^^,^^8^^)^ and kittens^(^^9^^)^, a moderate- or high-protein diet is considered to be beneficial in the treatment of cats with uroliths^(^^10^^)^.

A urinary pH < 6·29 is assumed to enhance the risk for the formation of CaOx uroliths in cats^(^^11^^,^^12^^)^, and the dietary protein concentration and source can modulate the pH of the urine. In one study, a high-protein diet (55 % crude protein (CP) on a DM basis), based on maize gluten meal, fish meal and soyabean meal, led to a urinary pH of 6·63 in cats, while feeding a moderate-protein diet (29 % CP on a DM basis) based on the same protein sources resulted in an alkaline urinary pH of 7·25^(^^13^^)^. Another study in cats found also an alkaline urinary pH (>7) when feeding diets with moderate protein concentrations (29–32 % CP on a DM basis) and meat meal, chicken meal or maize gluten meal as protein sources^(^^14^^)^. However, the highest urinary pH (7·99) was found when feeding the diet based on meat meal, and the lowest (7·08) when feeding the diet with maize gluten meal^(^^14^^)^. In human subjects, especially a high intake of animal protein has been shown to result in an acid load^(^^15^^)^.

Interestingly, little is known about the impact of dietary protein concentration on renal Ca excretion in cats. For human subjects, a high intake of animal protein or particularly of sulfur-containing amino acids can be considered to be critical for the formation of CaOx uroliths, since an increased renal Ca excretion is associated^(^^16^^,^^17^^)^. This increase is often explained to be a result of an acid load, derived from the oxidation of sulfur-containing amino acids to sulfuric acid and the associated release of protons^(^^18^^)^. Moreover, the acid load does not only increase the renal excretion of Ca, but also of uric acid, and decreases the renal excretion of citrate, all risk factors for the development of CaOx uroliths^(^^15^^)^. However, other authors found that the acid load may not solely be the reason for the increased renal Ca excretion associated with a high protein intake in human subjects^(^^19^^,^^20^^)^. In these studies, an increase in renal Ca excretion was observed even when adding the alkalising potassium bicarbonate or potassium citrate to a high-protein diet. Therefore, other factors may contribute to the increased renal Ca excretion, for instance a higher intestinal Ca absorption^(^^21^^,^^22^^)^. Moreover, since protein intake may modulate the secretion of PG^(^^23^^)^, an enhanced renal excretion of PGE_2_ could potentially regulate renal Ca excretion via the stimulation of calcitriol synthesis^(^^24^^,^^25^^)^.

Considering the sparse results on the relevance of dietary protein for the formation of CaOx uroliths in cats, the present study aimed to determine the effects of increasing dietary protein concentrations on feline urine composition in more detail. In addition, the impact of dietary protein quality has not been studied intensively. In one study by Zentek & Schulz^(^^2^^)^, the highest urinary Ox concentrations in cats were observed when feeding a low-protein diet (23 % CP on a DM basis) based on collagen tissue when compared with low- and high-protein diets (22–64 % CP on a DM basis) based on horse meat or soya protein isolate. This effect could possibly be explained by the higher concentrations of hydroxyproline and glycine in collagen tissue^(^^26^^)^, which are precursors for endogenous Ox synthesis^(^^6^^,^^27^^,^^28^^)^. Surprisingly, in the Zentek & Schulz study^(^^2^^)^, urinary Ox concentrations were lower when feeding a high-protein diet (78 % CP on a DM basis) based on collagen tissue when compared with the low-protein diet with collagen tissue as the protein source. Thus, the role of dietary protein quality for the formation of CaOx uroliths in cats needs further evaluation. The present study compared two diets with varying protein quality, characterised by different amounts of collagen-rich greaves meal in the diets. In contrast to the previous study^(^^2^^)^, the diets had comparable protein concentrations that allowed us to focus on the impact of protein quality as a single dietary factor on urine composition of cats.

## Materials and methods

### Animal study

The animal study was approved by the Animal Welfare Committee (Landesamt für Gesundheit und Soziales, Berlin, Germany, G 0004/08). A group of eight adult cats (European shorthair, four male, four female, aged 12–25 months) was included in the study. In total, four feeding periods were carried out, and each feeding period was divided into a 21 d adaptation period and a 7 d sampling period to allow for total urine and faeces collection. The cats were housed in groups (adaptation period) or individually (sampling period) in a room where a light (12 h light–12 h darkness) and temperature (20°C) regimen was kept constant at all times. All cats received socialisation throughout the study. The cats were fed once daily in the morning, while the constancy of body weight (BW) and the individual energy requirements of the cats were considered^(^^29^^)^. Water was provided *ad libitum*; however, since the water bowl for each cat was filled with 200 ml water per d, a measurement of the daily water intake was possible. The individual daily feed and water intake of the cats was documented during each sampling period.

### Diets, water and proximate analysis

Four extruded dry diets, varying in protein concentration and quality, were offered in four feeding periods. All cats were fed the same experimental diet at the same time (four-period, four-treatment parallel cross-over design).

The first three diets (HQ 35 %, HQ 44 % and HQ 57 %) were characterised by high protein quality (HQ) and differed in CP concentration (35, 44 and 57 % on a DM basis). The fourth diet (LQ 55 %) had a comparable CP concentration (55 % on a DM basis) as diet HQ 57 %, but lower protein quality (LQ). Protein quality was based on the amount of collagen-rich ingredients included in the experimental diets, which was 11 % greaves meal in diet HQ 57 % and 34 % greaves meal in diet LQ 55 %. The amount of greaves meal in the diets HQ 35 % and HQ 44 % was 10 % and therefore comparable with diet HQ 57 %.

All diets were formulated to fulfil the nutrient requirements of adult cats, according to the National Research Council^(^^29^^)^. The dietary components were selected to be low in Ox and the average dietary Ox concentrations were determined at 7 mg/100 g on a DM basis, representing an average value obtained from analysis of three diets. The Ca concentrations were also comparable in all diets (11·0–12·2 g/kg DM). The ingredients of the experimental diets and the results of the proximate analysis of the diets are presented in the Tables 1 and 2. The analysis was performed as described elsewhere^(^^30^^)^. In brief, the concentrations of crude nutrients were measured according to the directions of the Weende analysis of feed^(^^31^^)^, with a modified method for the determination of crude fat^(^^30^^)^. The mineral concentrations in the diets were measured as described below for minerals in the faeces of the cats. Ox concentrations in the diets were analysed using a commercial oxalate oxidase assay (Enzytec^TM^ Oxalsäure, no. E2100; R-Biopharm AG). For each feed sample, 1 g was mixed with 4 ml hydrochloric acid (5 m) for 15 min (Multi Reax; Heidolph Instruments GmbH & Co.) and subsequently heated at 60°C for 3 h (Haake C10-W13; Thermo Scientific). The samples were centrifuged at 2663 ***g*** and room temperature for 15 min (Heraeus Labofuge 400R; Thermo Scientific), and the aqueous phase was filtered (SCFA Syringe Filter, 0·2 µm; Thermo Scientific). A quantity of 500 µl of the filtered aqueous phase was adjusted to a pH of 2·9–3·1, using either hydrochloric acid or sodium hydroxide. Subsequently, the samples were centrifuged at 17 000 ***g*** and room temperature for 10 min (Heraeus Fresco 17 Centrifuge; Thermo Scientific). Then 10 µl of the supernatant fraction and 200 µl of Reagent 1 (Buffer, Enzytec^TM^; R-Biopharm AG) were mixed for 1 min at 1050 rpm and 37°C on an orbital shaker (BioShake iQ; Analytic Jena). After incubation for 5 min at 37°C and 590 nm (TECAN infinite M200 PRO; Tecan Group Ltd), 20 µl of Reagent 2 (oxalate oxidase, Enzytec^TM^; R-Biopharm) were added. The samples were mixed for 1 min at 1050 rpm and 37°C on an orbital shaker (BioShake iQ; Analytic Jena) and subsequently incubated at 37°C and 590 nm (TECAN infinite M200 PRO; Tecan Group Ltd). After 15 min, extinctions were measured (TECAN infinite M200 PRO; Tecan Group Ltd), and Ox concentrations were calculated according to the instructions of the manufacturer (Enzytec^TM^; R-Biopharm).

**Table 1.** Ingredients (%) of the experimental diets*

	High quality			Low quality
Analysed composition	HQ 35 %	HQ 44 %	HQ 57 %	LQ 55 %
Maize	44·2	32·7	9·43	19·2
Poultry meal	17·2	27·5	33·7	31·5
Soya protein	0·00	0·00	12·7	0·00
Maize gluten	9·51	11·6	17·0	0·00
Greaves meal	9·51	9·64	11·3	33·6
Pig fat	7·45	6·59	9·23	8·92
Rice gluten	2·85	2·89	0·00	0·00
Dried beet pulp	1·90	1·93	0·00	0·00
Digest and antioxidants	3·93	3·83	3·85	3·85
Salt	0·91	0·86	0·90	0·58
Yeast	0·95	0·96	0·94	0·96
Marigold meal	0·03	0·03	0·03	0·03
Minerals and vitamins	1·56	1·47	0·92	1·36

HQ 35 %, high protein quality diet with 35 % crude protein content; HQ 44 %, high protein quality diet with 44 % crude protein content; HQ 57 %, high protein quality diet with 57 % crude protein content; LQ 55 %, low protein quality diet with 55 % crude protein content.

* The quality differed depending on the amounts of collagen tissue in the diets: HQ 35 % and HQ 44 %, 10 % greaves meal; HQ 57 %, 11 % greaves meal; LQ 55 %, 34 % greaves meal.

**Table 2.** Nutrient analysis of the experimental diets*

	High quality			Low quality
Analysed composition	HQ 35 %	HQ 44 %	HQ 57 %	LQ 55 %
DM (g/kg)	904	919	923	928
Crude protein (g/kg DM)	347	438	574	547
Crude fat (g/kg DM)	115	124	171	183
Crude fibre (g/kg DM)	11·1	22·8	10·8	11·9
Crude ash (g/kg DM)	72·8	67·7	71·4	72·3
Ca (g/kg DM)	12·2	11·3	11·4	11·0
P (g/kg DM)	11·3	11·0	12·3	11·4
Na (g/kg DM)	7·40	6·51	7·52	7·72
K (g/kg DM)	6·30	5·24	7·36	6·97
Cl (g/kg DM)	11·6	9·50	8·34	10·7
Mg (g/kg DM)	1·08	1·12	1·36	0·89
ME (MJ/kg DM)†	17·0	17·2	18·6	18·8
NFE (g/kg DM)‡	454	348	173	186

HQ 35 %, high protein quality diet with 35 % crude protein content; HQ 44 %, high protein quality diet with 44 % crude protein content; HQ 57 %, high protein quality diet with 57 % crude protein content; LQ 55 %, low protein quality diet with 55 % crude protein content; ME, metabolisable energy; NFE, N-free extracts.

* The quality differed depending on the amounts of collagen tissue in the diets: HQ 35 % and HQ 44 %, 10 % greaves meal; HQ 57 %, 11 % greaves meal; LQ 55 %, 34 % greaves meal.

† Calculated according to the National Research Council^(^^29^^)^ using the four-step calculation with due regard to the crude fibre concentrations of the diets.

‡ Calculated as follows: NFE = DM – (crude ash + crude protein + crude fat + crude fibre).

For the analysis of drinking water, the water sample was mixed with a specific buffer, where the buffer varied depending on the analysed minerals. For the measurement of Zn and Cu, 1 ml water was mixed with 1 ml of a buffer that was based on 10 ml HCl (37 %) and 990 ml ultra-pure water. For the measurement of Ca concentration, 0·05 ml of the water sample and 4·95 ml of a buffer (10 ml HCl (37 %), 20 ml caesium chloride–lanthanum chloride buffer, 970 ml ultra-pure water) were mixed. The caesium chloride buffer was based on 10 g caesium chloride/l and 100 g lanthanum/l (Merck KGaA). For the determination of Na concentration in the water, 0·025 ml of the water sample and 4·975 ml of a buffer (10 ml HCl (37 %), 50 ml caesium chloride–lanthanum chloride buffer, 940 ml ultra-pure water) were mixed. The sample for the K measurement was prepared using 0·05 ml water and 4·95 ml of a buffer (10 ml HCl (37 %), 50 ml caesium chloride–lanthanum chloride buffer, 940 ml ultra-pure water). For the determination of Mg concentration, 1 ml of the water sample and 4 ml of a buffer (10 ml HCl (37 %), 1 g potassium chloride, 990 ml ultra-pure water) were mixed. Fe concentration was measured using a mixture of 1 ml water and 1 ml of a buffer (10 ml HCl (37 %), 1 g potassium chloride, 990 ml ultra-pure water).

The concentrations of Zn, Cu, Ca, Na, K, Mg and Fe in the prepared water samples were measured using atomic absorption spectrometry. For this, a flame atomic absorption spectrometer (type contra 700) with an autosampler (AS 52S) was used (Analytik Jena AG). For the analysis of P concentration in the water, 0·5 ml of the water sample, 1·5 ml ultra-pure water and 0·5 ml of colour reagent (nitro-vanadate–molybdate solution) were mixed. After 10 min, P concentration was measured spectrophotometrically^(^^32^^)^. The extinctions were determined with an Ultrospec 2100 pro Classic (Pharmacia Biotech) at a wavelength of 436 nm.

### Collection, preparation and analysis of the urine and faeces

During each sampling period, purpose-built cat litter boxes were used with plastic pellets as litter and connected urine collection containers to separate the urine from the faeces. Each urine collection container was provided with one drop of chlorhexidine digluconate to prevent bacterial growth in the urine. The cat litter boxes were checked for fresh urine and faeces three times per d, and the samples were stored at 4°C until the evening. Urinary pH was measured in the evening with the Seven Multi pH meter (Mettler-Toledo GmbH). After pH measurement, all urine and faeces samples of each day were stored at –80°C (urine) or –20°C (faeces) until further analysis. Sample preparation and analysis were as described elsewhere^(^^33^^)^. In short, the concentrations of urinary anions (sulfate and phosphate as major anions; Ox and citrate as minor anions) were measured with an ion exchange HPLC system (Dionex DX-500; Dionex Corp.), and also the concentrations of urinary cations (Na and K as major cations; Mg and Ca as minor cations) (Dionex DX-120). Data on the concentrations of urinary anions and cations were analysed using Chromeleon Client, version 6.80 SP2 (Dionex Corp.). The Cl concentrations in the faeces of the cats were determined using an ion exchange HPLC system (Dionex DX-500; Dionex Corp.). Faecal P concentrations were measured spectrophotometrically^(^^32^^)^ (Ultrospec 2000, Pharmacia Biotech), and the concentrations of Ca, Na, K and Mg in the faeces were measured using atomic absorption spectrometry (flame atomic absorption spectrometer type vario 6 with an autosampler AS 52; Analytik Jena AG). The urinary CaOx relative supersaturation (RSS CaOx) was calculated using the Supersat Program^(^^34^^)^.

### Renal and faecal excretion, apparent digestibility and retention

Renal excretion of the anions and cations was calculated as follows:

Renal excretion (mg/kg BW per d) = (anion or cation concentration in the urine (mg/ml) × total urinary volume (ml/d))/BW (kg).

Faecal excretion of the minerals was calculated as follows:

Faecal excretion (mg/kg BW per d) = (mineral concentration in the faeces (mg/g DM) × total amount of faeces (g DM/d))/BW (kg).

The apparent digestibility of the minerals was calculated with:

Apparent digestibility (%) = (mineral intake (mg/d) – faecal mineral excretion (mg/d))/mineral intake (mg/d) × 100.

Apparent digestibility of CP was calculated by analogy.

Mineral retention was calculated as follows:

Mineral retention (mg/d) = mineral intake (mg/d) – renal mineral excretion (mg/d) – faecal mineral excretion (mg/d).

### Statistical analysis

For the statistical analysis, the impact of a varying dietary protein concentration (diets HQ 35 %, HQ 44 %, HQ 57 %) and of a varying dietary protein quality (diet HQ 57 % *v.* diet LQ 55 %) was separately evaluated. For this, SPSS 15 (SPSS Inc.) was used. A repeated-measures ANOVA was performed (fixed factor protein concentration or protein quality), and within-subject contrasts (simple contrasts; protein concentration = three levels and protein quality = two levels; three tests per parameter for protein concentration and one test per parameter for protein quality) were considered for the detection of group differences. The data are presented in tables as mean values with their standard errors. Significant differences (*P* ≤ 0·05) among groups (separate for the comparisons of dietary protein concentration and dietary protein quality) are shown.

## Results

### Composition of drinking water

The analysed mineral concentrations in the drinking water were: 106 mg Ca/l, 37·8 mg Na/l, 10·9 mg K/l, 8·80 mg Mg/l, 0·58 mg Cu/l, 0·03 mg Zn/l and 0·01 mg Fe/l. The P concentration in the water was below the detection limit (1·33 mg/l).

### Animal health, body weight, feed and water intake, urinary volume and urinary pH

All cats were healthy throughout the study. The BW of the cats did not differ when receiving diets with different protein quality (HQ 57 % *v.* LQ 55 %), and showed small variations with differing dietary protein concentrations (HQ 35 %, HQ 44 %, HQ 57 %) (Table 3). The varying dietary protein quality had no impact on daily feed and water intake, urinary volume or urinary pH (Table 3). In contrast, increasing concentrations of dietary protein led to an increased urinary volume and a higher feed intake. Urinary pH was the lowest (6·34) after feeding diet HQ 44 % when compared with the diets HQ 35 % (6·66) and HQ 57 % (6·61).

**Table 3.** Body weight (BW), feed and water intake, urinary volume, urinary pH and urinary relative supersaturation with calcium oxalate (RSS CaOx) of cats fed a diet with a varying protein concentration and quality (Mean values with their standard errors; *n* 8 per diet)

	Protein concentration				Protein quality*		
	HQ 35 %	HQ 44 %	HQ 57 %	sem	HQ 57 %	LQ 55 %	sem
BW (kg)	4·07^b^	3·99^a^	4·17^b^	0·30	4·17	4·22	0·36
Feed intake (g DM/kg BW per d)	13·3^a^	13·3^a,b^	16·0^b^	0·83	16·0	13·4	1·03
Water intake (ml/kg BW per d)	29·8	29·8	35·4	1·79	35·4	32·5	1·77
Urinary volume (ml/kg BW per d)	9·86^a^	10·9^a^	17·0^b^	1·03	17·0	14·7	1·05
Urinary pH	6·66^b^	6·34^a^	6·61^b^	0·04	6·61	6·58	0·02
Urinary RSS CaOx	8·24^a^	10·9^b^	11·2^b^	0·80	11·2	10·1	0·94

HQ 35 %, high protein quality diet with 35 % crude protein content; HQ 44 %, high protein quality diet with 44 % crude protein content; HQ 57 %, high protein quality diet with 57 % crude protein content; LQ 55 %, low protein quality diet with 55 % crude protein content.

^a,b^ Mean values within a row with unlike superscript letters were significantly different (*P* ≤ 0·05).

* Protein quality differed depending on the amounts of greaves meal in the diets: HQ 35 % and HQ 44 %, 10 % greaves meal; HQ 57 %, 11 % greaves meal; LQ 55 %, 34 % greaves meal.

### Effect of dietary protein concentration

#### Impact on urinary concentration and excretion of calcium and oxalate and the urinary relative supersaturation with calcium oxalate

Urinary Ca concentrations and renal Ca excretion increased with increasing dietary protein concentrations (Table 4). Urinary Ox concentration was the lowest after feeding diet HQ 57 % when compared with the diets HQ 35 % and HQ 44 %; however, calculating renal Ox excretion demonstrated an increase from 1·08 mg/kg BW per d (HQ 35 %) to 1·68 mg/kg BW per d (HQ 57 %). Urinary RSS CaOx also increased with increasing protein concentrations in the diets, reaching values between 8·24 (HQ 35 %) and 11·2 (HQ 57 %) (Table 3).

**Table 4.** Urinary concentration and renal excretion of anions and cations of cats fed a diet with a varying protein concentration and quality (Mean values with their standard errors; *n* 8 per diet)

	Protein concentration				Protein quality†		
	HQ 35 %	HQ 44 %	HQ 57 %	sem	HQ 57 %	LQ 55 %	sem
Urinary concentration (mg/l)							
Ca	60·5^a^	59·4^a^	78·3^b^	4·32	78·3	68·0	5·16
P	2880	2658	2608	178	2608	2557	207
Mg	33·0^a^	47·6^b^	29·9^a^	3·08	29·9	25·0	3·25
K	2165^b^	2009^b^	1773^a^	107	1773	1994	124
Na	2443^b^	1990^a,b^	1947^a^	139	1947	2162	181
Ox	118^b^	135^b^	102^a^	7·53	102	104	5·72
Sulfate	3277	3664	3548	293	3548	2911*	317
Citrate	148^b^	65·0^a^	80·8^a^	16·8	80·8	67·0	12·2
Renal excretion (mg/kg BW per d)							
Ca	0·59^a^	0·69^a^	1·32^b^	0·10	1·32	0·98*	0·12
P	27·3^a^	30·3^a,b^	43·8^b^	3·06	43·8	34·7	3·16
Mg	0·30^a^	0·54^a,b^	0·50^b^	0·04	0·50	0·35	0·05
K	20·3^a^	22·2^a,b^	29·7^b^	1·91	29·7	28·2	2·23
Na	22·2^a^	21·6^a^	32·3^b^	1·65	32·3	29·7	2·32
Ox	1·08^b^	1·51^a,b^	1·68^a^	0·10	1·68	1·48	0·10
Sulfate	30·6^a^	43·5^a,b^	59·4^b^	5·18	59·4	39·0	5·50
Citrate	1·20^b^	0·64^a^	1·29^b^	0·13	1·29	0·89	0·16

HQ 35 %, high protein quality diet with 35 % crude protein content; HQ 44 %, high protein quality diet with 44 % crude protein content; HQ 57 %, high protein quality diet with 57 % crude protein content; LQ 55 %, low protein quality diet with 55 % crude protein content; BW, body weight.

^a,b^ Mean values within a row with unlike superscript letters were significantly different (*P* ≤ 0·05).

* Mean value was significantly different from that for HQ 57 % (*P* ≤ 0·05).

† Protein quality differed depending on the amounts of greaves meal in the diets: HQ 35 % and HQ 44 %, 10 % greaves meal; HQ 57 %, 11 % greaves meal; LQ 55 %, 34 % greaves meal.

#### Impact on urinary concentration and excretion of sodium, potassium, phosphorus, magnesium, citrate and sulfate

Urinary Na and K concentrations were the lowest in the group that received diet HQ 57 % when compared with the diets HQ 35 % and HQ 44 %; however, calculating the renal excretion of these minerals demonstrated contrary results (Table 4). While urinary P concentrations were unaffected by dietary protein concentrations, renal P excretion increased with increasing protein in the diets. Urinary Mg concentrations and renal Mg excretion varied among the groups, while no unidirectional effect of protein concentration in the diet could be observed. Urinary citrate concentrations were markedly higher after feeding diet HQ 35 % (148 mg/l) when compared with the other groups (65·0–80·8 mg/l); however, based on daily renal citrate excretion, the amounts were comparable between the groups HQ 35 % and HQ 57 %, but lower in group HQ 44 %. Urinary sulfate concentrations were not affected by the protein concentration in the diet, while renal sulfate excretion increased with increasing dietary protein concentrations.

#### Impact on amount and DM of the faeces, faecal mineral concentrations and excretion

The daily amount of faeces on a DM basis was the lowest in group HQ 44 % when compared with the other groups, and the DM concentration of the faeces decreased with increasing dietary protein (Table 5).

**Table 5.** Amount of faeces, DM of the faeces, mineral concentrations in the faeces and faecal mineral excretion of cats fed a diet with a varying protein concentration and quality (Mean values with their standard errors; *n* 8 per diet)

	Protein concentration				Protein quality†		
	HQ 35 %	HQ 44 %	HQ 57 %	sem	HQ 57 %	LQ 55 %	sem
Amount of faeces (g/kg BW per d)	5·25^a^	4·46^a^	7·35^b^	0·53	7·35	5·74	0·64
DM of the faeces (%)	44·2^a,b^	42·4^b^	39·3^a^	0·83	39·3	49·0	2·23
Amount of faeces (g DM/kg BW per d)	2·33^b^	1·85^a^	2·83^b^	0·19	2·83	2·88	0·31
Faecal concentrations (mg/g DM)							
Ca	72·1^b^	63·0^a^	77·2^b^	1·62	77·2	68·2*	2·06
P	48·8^b^	33·2^a^	50·9^b^	1·81	50·9	41·1*	1·44
Mg	6·10^b^	5·24^a^	7·12^b^	0·20	7·12	4·72*	0·35
K	2·16^a^	4·48^b^	2·36^a^	0·37	2·36	1·08*	0·28
Na	2·13^b^	1·98^a^	2·35^b^	0·13	2·35	2·21	0·15
Cl	1·50	1·64	1·78	0·21	1·78	1·35	0·27
Faecal excretion (mg/kg BW per d)							
Ca	167^b^	117^a^	224^c^	16·9	224	166*	21·5
P	112^b^	62·2^a^	144^b^	10·8	144	115	12·5
Na	5·27^a,b^	3·70^a^	6·93^b^	0·68	6·93	6·65	1·07
Mg	14·3^b^	9·76^a^	20·1^b^	1·49	20·1	13·2*	1·77
K	5·91	8·91	7·27	1·14	7·27	2·94*	1·10
Cl	3·69	3·32	5·58	0·77	5·58	4·11	1·12

HQ 35 %, high protein quality diet with 35 % crude protein content; HQ 44 %, high protein quality diet with 44 % crude protein content; HQ 57 %, high protein quality diet with 57 % crude protein content; LQ 55 %, low protein quality diet with 55 % crude protein content; BW, body weight.

^a,b,c^ Mean values within a row with unlike superscript letters were significantly different (*P* ≤ 0·05).

* Mean value was significantly different from that for HQ 57 % (*P* ≤ 0·05).

† Protein quality differed depending on the amounts of greaves meal in the diets: HQ 35 % and HQ 44 %, 10 % greaves meal; HQ 57 %, 11 % greaves meal; LQ 55 %, 34 % greaves meal.

The protein concentration of the diets affected mineral concentrations in the faeces of the cats; however, no unidirectional effect could be detected (Table 5). The lowest faecal Ca, P, Mg and Na concentrations, but the highest faecal K concentrations, were observed in group HQ 44 % when compared with the other groups. Cl concentrations in the faeces of the cats did not differ among the groups. The described observations also applied for faecal Ca, P, Mg and Na excretion, while faecal K and Cl excretion was unaffected by dietary protein concentration (Table 5).

#### Impact on apparent digestibility and mineral retention

The apparent digestibility of CP was the lowest in group HQ 35 % when compared with the groups HQ 44 % and HQ 57 % (Table 6). As described for the mineral concentrations in the faeces of the cats, no unidirectional effect of protein concentration in the diet could be observed for the apparent digestibility and the retention of the minerals. The highest apparent digestibility of Ca, P and Mg, but the lowest apparent digestibility of K, was observed in group HQ 44 % when compared with the other groups. The apparent digestibility of Na and Cl did not differ among the groups. The retention of Ca, P and Mg was the highest, and the retention of Na and K was the lowest, in group HQ 44 % when compared with the groups HQ 35 % and HQ 57 % (Table 6).

**Table 6.** Apparent digestibility of crude protein (CP) and minerals, and mineral retention of cats fed a diet with a varying protein concentration and quality (Mean values with their standard errors; *n* 8 per diet)

	Protein concentration				Protein quality†		
	HQ 35 %	HQ 44 %	HQ 57 %	sem	HQ 57 %	LQ 55 %	sem
Apparent digestibility (%)							
CP	83·3^a^	88·7^b^	87·6^b^	0·80	87·6	82·9	1·60
Ca	0·09^a^	22·5^b^	–19·1^a^	5·46	–19·1	–16·2	5·46
P	27·1^a^	57·6^b^	27·2^a^	3·90	27·2	23·5	3·90
Na	95·0	95·7	94·3	0·42	94·3	93·6	0·42
Mg	4·73^a^	34·4^b^	7·60^a^	4·68	7·60	–11·1	4·68
K	93·6^b^	87·5^a^	94·1^b^	1·29	94·1	96·5	1·29
Cl	97·7	97·4	96·0	0·51	96·0	97·2	0·51
Retention (mg/d)							
Ca	–4·92^a^	98·8^b^	–32·6^a,b^	43·3	–32·6	–81·0	69·1
P	43·8^a^	172^b^	28·1^a^	23·7	28·1	11·1	32·7
Na	267^b^	213^a^	313^c^	15·5	313	269	19·4
Mg	0·90^a^	14·2^b^	4·20^a,b^	2·90	4·20	–4·72	3·99
K	217^b^	131^a^	313^c^	18·7	313	247*	20·0

HQ 35 %, high protein quality diet with 35 % crude protein content; HQ 44 %, high protein quality diet with 44 % crude protein content; HQ 57 %, high protein quality diet with 57 % crude protein content; LQ 55 %, low protein quality diet with 55 % crude protein content.

^a,b,c^ Mean values within a row with unlike superscript letters were significantly different (*P* ≤ 0·05).

* Mean value was significantly different from that for HQ 57 % (*P* ≤ 0·05).

† Protein quality differed depending on the amounts of greaves meal in the diets: HQ 35 % and HQ 44 %, 10 % greaves meal; HQ 57 %, 11 % greaves meal; LQ 55 %, 34 % greaves meal.

### Effect of dietary protein quality

#### Impact on urinary concentration and excretion of calcium and oxalate and urinary relative supersaturation with calcium oxalate

The protein quality of the diets did not influence the total concentrations of Ca and Ox in the urine of the cats, renal Ox excretion or urinary RSS CaOx values (Tables 3 and 4). However, renal Ca excretion was higher when feeding the diet with the lower amounts of greaves meal (HQ 57 %).

#### Impact on urinary concentration and excretion of sodium, potassium, phosphorus, magnesium, citrate and sulfate

The protein quality of the diets did not affect the concentrations of P, Mg, Na and K in the urine of the cats or the renal excretion of these minerals (Table 4). Urinary sulfate concentrations were lower in group LQ 55 % when compared with group HQ 57 %; however, renal sulfate excretion did not differ between the groups. Urinary citrate concentrations and renal citrate excretion were not affected by a different protein quality of the diets.

#### Impact on amount and DM of faeces, faecal mineral concentrations and excretion

The protein quality of the diets did not affect the daily amount of faeces of the cats or the faecal DM concentration (Table 5). Faecal Ca, P, Mg and K concentrations were lower in group LQ 55 % when compared with group HQ 57 %, while this effect was also observed for faecal Ca, Mg and K excretion (Table 5). Faecal Na and Cl concentrations and excretion were not affected by the varying amount of greaves meal in the diets.

#### Impact on apparent digestibility and mineral retention

The apparent digestibility of CP was approximately 5 % lower in group LQ 55 % when compared with group HQ 57 %; however, this difference was not statistically significant (*P* > 0·05) (Table 6). The protein quality of the diets did not affect the apparent digestibility or the retention of the minerals (Table 6). The only exception was K retention, which was higher in group HQ 57 % when compared with group LQ 55 %.

## Discussion

The present study was conducted to investigate the hypothesis that a high-protein diet can be considered as a risk factor for the formation of CaOx uroliths in cats. While this is the scientific consensus in human subjects, since an increase in renal Ca and Ox excretion has been associated with a high protein intake^(^^1^^,^^17^^)^, previous studies have indicated that a high-protein diet could be beneficial for the prevention of CaOx uroliths in cats^(^^2^^,^^3^^,^^10^^,^^35^^)^. However, these studies were based on an epidemiological risk evaluation^(^^35^^)^ or have only focused on the effects of dietary protein on the urinary volume^(^^10^^)^ and urinary Ox excretion^(^^2^^,^^3^^)^, while other risk factors for the formation of CaOx uroliths were not considered.

Urinary Ox concentrations in the present study were the lowest when feeding the high-protein diet (HQ 57 %); however, no strict decrease with increasing dietary protein concentrations could be detected, since the group that received the medium-protein diet (HQ 44 %) showed the highest urinary Ox concentrations and the difference between the low (HQ 35 %)- and high-protein group was small. In contrast, a unidirectional increase in renal Ox excretion was observed with increasing dietary protein concentrations. It can be assumed that the lower urinary Ox concentrations did result from the increase in the urinary volume associated with the high-protein diet. In general, it could be supposed that a lower urinary Ox concentration is of higher relevance than the increase in renal Ox excretion with regard to CaOx urolith formation. However, in this context, the calculated RSS CaOx values should not go unmentioned. We observed an increase in urinary RSS CaOx with increasing dietary protein concentrations, indicating that a high-protein diet may be critical for the formation of CaOx uroliths in cats and that the increase in renal Ox excretion with increasing dietary protein concentrations should not be undervalued.

The increased renal Ox excretion can possibly be explained by an enhanced endogenous Ox synthesis. In general, the amounts of Ox in cat food are considered to be small^(^^6^^)^ and the average Ox concentration in the present diets was determined at 7 mg/100 g DM. Food components that are rich in Ox include vegetables and grain, moderate Ox concentrations are found in cereals and nuts, and only low amounts in food of animal origin like meat, milk products and fish^(^^36^^,^^37^^)^. In a previous study^(^^3^^)^, renal Ox excretion was not affected by a high-protein diet based on casein. However, the authors concluded that this effect may be different in the case of another protein source. In the present study, the main protein source was poultry meal. Since poultry meal is often used for commercial cat food, the present results are of high practical relevance.

Interestingly, protein quality did not affect urinary Ox concentrations or renal Ox excretion. It was hypothesised that a higher amount of hydroxyproline and glycine, derived from collagen-rich greaves meal in the diets, would enhance endogenous Ox synthesis. Another study demonstrated that a diet with collagen tissue as the protein source led to higher urinary Ox excretion when compared with diets based on horse meat or soya protein isolate^(^^2^^)^. However, the authors also demonstrated that the diet with lower amounts of collagen tissue resulted in higher urinary Ox excretion than a diet with high amounts of collagen tissue. This observation might indicate that other dietary factors than specific amino acids derived from collagen tissue could be important for endogenous Ox synthesis. In particular, one factor could be a higher fat concentration in the diet. In this previous study^(^^2^^)^, the fat concentration in the diet with low amounts of collagen tissue was 24 % on a DM basis, but in the diet with high amounts of collagen tissue 10 %, and it could be hypothesised that a higher dietary fat concentration enhanced endogenous Ox synthesis. Recent results^(^^3^^)^ support this assumption, as higher urinary Ox concentrations were measured when feeding a high-fat diet compared with a high-protein diet. Up to now, the potential mechanisms that lead to an increased renal Ox excretion by a high fat intake remain unclear. It is hypothesised that high dietary fat concentrations could contribute to a complexation between fatty acids and Ca in the intestine. This would reduce the available amounts of Ca for binding Ox and increase the intestinal Ox absorption and renal Ox excretion^(^^38^^)^. Schmiedl *et al.*^(^^39^^)^ suggest that hyperlipidaemia could increase hepatic Ox synthesis. The rats of this study showed hyperlipidaemia and elevated concentrations of lactate dehydrogenase (LDH) in the blood when fed a high-fat diet. LDH can synthesise Ox from glycolate or other Ox precursors. Finally, the role of band 3 protein is discussed with regard to renal Ox excretion^(^^38^^)^. Band 3 protein is an anion transporter that catalyses the exchange of anions (for example, Ox) across a cell membrane. An activation (phosphorylation) of band 3 protein in erythrocytes in the intestine and kidneys can therefore increase intestinal Ox absorption and renal Ox excretion^(^^38^^)^. It has been demonstrated that arachidonic acid leads to a phosphorylation of band 3 protein in a dose-dependent manner^(^^40^^)^.

It should be taken into consideration that the diets HQ 35 %, HQ 44 % and HQ 57 % showed some variations in fat concentrations (115, 124 and 171 g/kg DM, respectively). Thus, it cannot be excluded that the observed increase in renal Ox excretion associated with the high-protein diet could partly derive from the increase in dietary fat concentration. However, the diets HQ 57 % and LQ 55 % had comparable fat concentrations, independently of the amount of greaves meal. This aspect may be one reason for the observed similar urinary Ox concentrations and renal Ox excretion in these two groups. The present results highlight that a higher dietary amount of certain amino acids as potential precursors of endogenous Ox does not enhance endogenous Ox synthesis and may therefore be singularly no specific risk factor for the formation of CaOx uroliths in cats. In this context, it should not go unmentioned that the diets HQ 57 % and LQ 55 % varied in Mg concentrations (1·36 and 0·89 g/kg DM). High amounts of Mg can form a complex with Ox in the intestine, resulting in reduced intestinal absorption and renal excretion of Ox^(^^41^^)^. However, since no differences in urinary Ox concentrations or renal Ox excretion were observed between the two treatment groups, this effect seems to be negligible for the present study.

Urinary Ca concentrations and renal Ca excretion increased with increasing protein concentrations in the present experimental diets. This observation is consistent with findings in human subjects^(^^17^^)^. However, the main explanation for the increase in renal Ca excretion by a high protein intake is an acid load derived from the oxidation of sulfur-containing amino acids to sulfuric acid and the associated release of protons^(^^18^^)^. In the present study, urinary pH did not decrease with increasing concentrations of dietary protein, indicating that the enhanced renal Ca excretion was not mediated by an acid load. Nevertheless, although urinary sulfate concentrations were also unaffected by increasing dietary protein concentrations, daily renal sulfate excretion doubled from 30·6 mg/kg BW to 59·4 mg/kg BW, indicating an enhanced oxidation of sulfur-containing amino acids when feeding the high-protein diet. Another theory implies that a high-protein diet could enhance intestinal Ca absorption and consecutively also renal Ca excretion^(^^21^^,^^22^^)^. The present results did not demonstrate an increased apparent digestibility of Ca, but a higher faecal Ca excretion when feeding the high-protein diet. Since renal Ca excretion also increased, a marked negative Ca balance was observed. It can therefore be hypothesised that the increase in urinary Ca concentrations and renal Ca excretion with increasing dietary protein concentrations did not derive from a higher intestinal Ca absorption, but potentially from a Ca mobilisation within the organism. A negative Ca balance associated with a high protein intake has also been observed in young men^(^^42^^)^, and a negative correlation between protein intake and bone mineral content as well as between protein intake and bone density has been identified in young women^(^^43^^)^. Future studies should therefore evaluate whether the demonstrated negative Ca balance associated with a high-protein diet also affects bone mass in cats.

With regard to the increase in urinary Ca concentrations and renal Ca excretion with increasing dietary protein concentrations, it should be finally mentioned that the crude fibre concentrations of the diets HQ 35 %, HQ 44 % and HQ 57 % were relatively low, but showed some variation (11·1, 22·8 and 10·8 g/kg DM, respectively). In our experience, the analysed crude fibre concentrations in cat food can vary considerably, which requires a careful interpretation of the results. Extremely high dietary fibre concentrations (11–14 % in DM) have been reported to be associated with an increase in faecal DM excretion in cats, and faecal Ca excretion is positively correlated with faecal DM excretion^(^^44^^)^. These observations may indicate a reduction in intestinal Ca absorption by high dietary fibre concentrations, which has also been observed in rats when feeding diets with 2·62–3·25 % crude fibre on a DM basis compared with a control diet with 0·77 % crude fibre in DM^(^^45^^)^. As the present study demonstrated an increase in urinary Ca concentrations and renal Ca excretion, and no decrease was observed when feeding diet HQ 44 %, an effect on intestinal Ca absorption by varying crude fibre concentrations in the diets can be excluded.

Interestingly, renal Ca excretion was lower when feeding the diet with the higher amount of greaves meal (LQ 55 %). Up to now, the reason for this effect has remained unclear. Compared with diet LQ 55 %, diet HQ 57 % contained less greaves meal and a mixture of poultry meal and soya protein. This mixture has ensured similar mineral and, with the exception of CP in the HQ groups, similar macronutrient concentrations compared with the other experimental diets. The apparent digestibility of CP tended to be higher in group HQ 57 % compared with group LQ 55 %, which might have influenced the amino acid, mineral and acid base metabolism of the cats. It could be demonstrated that the urinary sulfate concentrations (*P* ≤ 0·05), and the daily renal sulfate excretion (*P* > 0·05) were higher when feeding diet HQ 57 % compared with diet LQ 55 %. However, since urinary pH was unaffected by protein quality in the diets, an acid load that enhanced renal Ca excretion when feeding diet HQ 57 % could not be clearly detected. Thus, the potential impact of dietary protein quality on renal Ca excretion in cats cannot be conclusively clarified by the present study and needs further evaluation.

Urinary volume increased with higher protein concentrations in the diets. This finding is in accordance with previous studies in adult cats and kittens^(^^7^^–^^9^^)^. A higher dilution of the urine can generally be advantageous for the prevention of urinary crystals and stones^(^^10^^)^. However, since the higher dietary protein concentrations were also associated with an increased renal Ca and Ox excretion, the higher urinary volume seems to be of minor importance. Dietary protein quality did not affect the urinary volume of the cats. Urinary pH was relatively low among all experimental groups, reaching values between 6·34 and 6·66. Since a urinary pH < 6·29 is considered to be a risk factor for the development of CaOx uroliths in cats^(^^11^^,^^12^^)^, the measured values were near to this critical range.

For the interpretation of the present results, limitations of the study design should finally not go unmentioned. In the present study, a four-period, four-treatment parallel cross-over design was used, which cannot eliminate carryover effects. However, the adaptation period was relatively long, and the cats were housed in a room with a constant light and temperature regimen; therefore constant conditions can reasonably be assumed for the present study.

In conclusion, the present data indicate that a high-protein diet cannot be considered as beneficial for the prevention of CaOx uroliths in cats, as previously assumed. Although a higher urinary volume was also associated with a higher protein intake, the increased urinary Ca concentrations, renal Ca and Ox excretion and urinary RSS CaOx values are critical and potential risk factors for the formation of CaOx uroliths. In addition, the clinical relevance of the demonstrated negative Ca balance when feeding a high-protein diet should be further evaluated with regard to bone turnover. The impact of dietary protein quality on urine composition was generally low, with no effect on urinary Ca and Ox concentrations, RSS CaOx or renal Ox excretion. However, the observed lower renal Ca excretion in cats that received a diet with a higher amount of collagen-rich greaves meal needs further investigation.

## References

[1] Nguyen QV, Kalin A, Drouve U, (2001) Sensitivity to meat protein intake and hyperoxaluria in idiopathic calcium stone formers. Kidney Int 59, 2273–2281. 11380831 10.1046/j.1523-1755.2001.00744.x

[2] Zentek J & Schulz A (2004) Urinary composition of cats is affected by the source of dietary protein. J Nutr 134, 2162S–2165S. 15284427 10.1093/jn/134.8.2162S

[3] Dijcker JC, Hagen-Plantinga EA & Hendriks WH (2012) Changes in dietary macronutrient profile do not appear to affect endogenous urinary oxalate excretion in healthy adult cats. Vet J 194, 235–239. 22578635 10.1016/j.tvjl.2012.03.029

[4] Masai M, Ito H & Kotake T (1995) Effect of dietary intake on urinary oxalate excretion in calcium oxalate stone formers. Br J Urol 76, 692–696. 8535710 10.1111/j.1464-410x.1995.tb00758.x

[5] Naya Y, Ito H, Masai M, (2000) Effect of dietary intake on urinary oxalate excretion in calcium oxalate stone formers in their forties. Eur Urol 37, 140–144. 10705190 10.1159/000020130

[6] Dijcker JC, Plantinga EA, van Baal J, (2011) Influence of nutrition on feline calcium oxalate urolithiasis with emphasis on endogenous oxalate synthesis. Nutr Res Rev 24, 96–110. 21338551 10.1017/S0954422410000351

[7] Hashimoto M, Funaba M, Abe M, (1995) Dietary protein levels affect water intake and urinary excretion of magnesium and phosphorus in laboratory cats. Exp Anim 44, 29–35. 7705476 10.1538/expanim.44.29

[8] Funaba M, Hashimoto M, Yamanaka C, (1996) Effects of a high-protein diet on mineral metabolism and struvite activity product in clinically normal cats. Am J Vet Res 57, 1726–1732. 8950426

[9] Hashimoto M, Funaba M, Abe M, (1996) Effect of chronic high protein intake on magnesium, calcium, and phosphorus balance in growing cats. Exp Anim 45, 63–70. 8689582 10.1538/expanim.45.63

[10] Kerr KR (2013) Companion Animals Symposium: dietary management of feline lower urinary tract symptoms. J Anim Sci 91, 2965–2975. 23408812 10.2527/jas.2012-6035

[11] Kirk CA, Ling GV, Franti CE, (1995) Evaluation of factors associated with development of calcium oxalate urolithiasis in cats. J Am Vet Med Assoc 207, 1429–1434. 7493870

[12] Osborne CA, Lulich JP, Thumchai R, (1995) Etiopathogenesis and therapy of feline calcium oxalate urolithiasis. In *Proceedings of the 13th Annual ACVIM Forum*, *Orlando, FL*, pp. 487–489. Lakewoood, CO: American College of Veterinary Internal Medicine (ACVIM).

[13] Funaba M, Yamate T, Hashida Y, (2003) Effects of a high-protein diet versus dietary supplementation with ammonium chloride on struvite crystal formation in urine of clinically normal cats. Am J Vet Res 64, 1059–1064. 12926602 10.2460/ajvr.2003.64.1059

[14] Funaba M, Oka Y, Kobayashi S, (2005) Evaluation of meat meal, chicken meal, and corn gluten meal as dietary sources of protein in dry cat food. Can J Vet Res 69, 299–304. 16479729 PMC1250243

[15] Negri AL, Spivacow FR & Del Valle EE (2013) Diet in the treatment of renal lithiasis. Pathophysiological basis. Medicina (B Aires) 73, 267–271. 23732207

[16] Zemel MB, Schuette SA, Hegsted M, (1981) Role of the sulfur-containing amino acids in protein-induced hypercalciuria in men. J Nutr 111, 545–552. 7205407 10.1093/jn/111.3.545

[17] Bihuniak JD, Simpson CA, Sullivan RR, (2013) Dietary protein-induced increases in urinary calcium are accompanied by similar increases in urinary nitrogen and urinary urea: a controlled clinical trial. J Acad Nutr Diet 113, 447–451. 23438496 10.1016/j.jand.2012.11.002 PMC5868414

[18] Sabry ZI, Shadarevian SB, Cowan JW, (1965) Relationship of dietary intake of sulphur amino-acids to urinary excretion of inorganic sulphate in man. Nature 206, 931–933. 5839851 10.1038/206931b0

[19] Ceglia L, Harris SS, Abrams SA, (2009) Potassium bicarbonate attenuates the urinary nitrogen excretion that accompanies an increase in dietary protein and may promote calcium absorption. J Clin Endocrinol Metab 94, 645–653. 19050051 10.1210/jc.2008-1796 PMC2730228

[20] Maalouf NM, Moe OW, Adams-Huet B, (2011) Hypercalciuria associated with high dietary protein intake is not due to acid load. J Clin Endocrinol Metab 96, 3733–3740. 21976719 10.1210/jc.2011-1531 PMC3232614

[21] Kerstetter JE, O'Brien KO, Caseria DM, (2005) The impact of dietary protein on calcium absorption and kinetic measures of bone turnover in women. J Clin Endocrinol Metab 90, 26–31. 15546911 10.1210/jc.2004-0179

[22] Hunt JR, Johnson LK & Fariba Roughead ZK (2009) Dietary protein and calcium interact to influence calcium retention: a controlled feeding study. Am J Clin Nutr 89, 1357–1365. 19279077 10.3945/ajcn.2008.27238

[23] Piccoli A, Calò L, Modena F, (1991) Prostaglandins and renal response to protein loading in normal and glomerulonephritic kidneys: effect of indomethacin and dipyridamole. Curr Ther Res Clin Exp 49, 596–609.

[24] Calò L, Cantaro S, Marchini F, (1990) Is hydrochlorothiazide-induced hypocalciuria due to inhibition of prostaglandin E_2_ synthesis? Clin Sci 78, 321–325. 2156653 10.1042/cs0780321

[25] Baggio B (2004) Protein diet and hypercalciuria. Kidney Int 65, 1970; author reply 1970. 10.1111/j.1523-1755.2004.607_1.x 15086945

[26] Laser Reutersward A, Asp NG & Bjork I (1985) Protein digestibility of pigskin and bovine tendon in rats. J Food Technol 20, 745–752.

[27] Knight J, Jiang J, Assimos DG, (2006) Hydroxyproline ingestion and urinary oxalate and glycolate excretion. Kidney Int 70, 1929–1934. 17021603 10.1038/sj.ki.5001906 PMC2268952

[28] Knight J, Easter LH, Neiberg R, (2009) Increased protein intake on controlled oxalate diets does not increase urinary oxalate excretion. Urol Res 37, 63–68. 19183980 10.1007/s00240-009-0170-z PMC2683385

[29] National Research Council (2006) Nutrient Requirements of Dogs and Cats. Washington, DC: The National Academies Press.

[30] Paßlack N, Brenten T, Neumann K, (2014) Investigations on the effects of potassium chloride and potassium bicarbonate in the diet on the urinary pH and mineral excretion of adult cats. Br J Nutr 111, 785–797. 24229496 10.1017/S0007114513003279

[31] Naumann C & Bassler C (2004) Die chemische Untersuchung von Futtermitteln 3. Aufl., 5. Ergänzungslieferung (Chemical Feed Analyses, Vol. 3). Darmstadt: VDLUFA-Verlag.

[32] Passlack N & Zentek J (2013) Urinary calcium and oxalate excretion in healthy adult cats are not affected by increasing dietary calcium levels. PLOS ONE 8, e51. 10.1371/journal.pone.0070530 PMC3734279 23940588

[33] Gericke S & Kurmies B (1952) Colorimetrische Bestimmung der Phosphorsäure mit Vanadat-Molybdat (Colorimetric determination of phosphoric acid with vanadate molybdate). Fres Zeitsch Anal Chem 137, 15–22.

[34] Robertson WG, Jones JS, Heaton MA, (2002) Predicting the crystallization potential of urine from cats and dogs with respect to calcium oxalate and magnesium ammonium phosphate (struvite). J Nutr 132, 1637S–1641S. 12042478 10.1093/jn/132.6.1637S

[35] Lekcharoensuk C, Osborne CA, Lulich JP, (2001) Association between dietary factors and calcium oxalate and magnesium ammonium phosphate urolithiasis in cats. J Am Vet Med Assoc 219, 1228–1237. 11697365 10.2460/javma.2001.219.1228

[36] Siener R, Honow R, Voss S, (2006) Oxalate content of cereals and cereal products. J Agric Food Chem 54, 3008–3011. 16608223 10.1021/jf052776v

[37] Massey LK (2007) Food oxalate: factors affecting measurement, biological variation, and bioavailability. J Am Diet Assoc 107, 1191–1194. 17604750 10.1016/j.jada.2007.04.007

[38] Naya Y, Ito H, Masai M, (2002) Association of dietary fatty acids with urinary oxalate excretion in calcium oxalate stone-formers in their fourth decade. BJU Int 89, 842–846. 12010225 10.1046/j.1464-410x.2002.02740.x

[39] Schmiedl A, Schwille PO, Bonucci E, (2000) Nephrocalcinosis and hyperlipidemia in rats fed a cholesterol- and fat-rich diet: association with hyperoxaluria, altered kidney and bone minerals, and renal tissue phospholipidcalcium interaction. Urol Res 28, 404–415. 11221920 10.1007/s002400000144

[40] Baggio B, Priante G, Brunati AM, (1999) Specific modulatory effect of arachidonic acid on human red blood cell oxalate transport: clinical implications in calcium oxalate nephrolithiasis. J Am Soc Nephrol 10, S381–S384. 10541268

[41] Morozumi M, Hossain RZ, Yamakawa K, (2006) Gastrointestinal oxalic acid absorption in calcium-treated rats. Urol Res 34, 168–172. 16705467 10.1007/s00240-006-0035-7

[42] Hegsted M, Schuette SA, Zemel MB, (1981) Urinary calcium and calcium balance in young men as affected by level of protein and phosphorus intake. J Nutr 111, 553–562. 7205408 10.1093/jn/111.3.553

[43] Metz JA, Anderson JJ & Gallagher PN Jr (1993) Intakes of calcium, phosphorus, and protein, and physical-activity level are related to radial bone mass in young adult women. Am J Clin Nutr 58, 537–542. 8379510 10.1093/ajcn/58.4.537

[44] Prola L, Dobenecker B, Mussa PP, (2010) Influence of cellulose fibre length on faecal quality, mineral excretion and nutrient digestibility in cat. J Anim Physiol Anim Nutr (Berl) 94, 362–367. 19663982 10.1111/j.1439-0396.2008.00916.x

[45] Gralak MA, Leontowicz M, Morawiec M, (1996) Comparison of the influence of dietary fibre sources with different proportions of soluble and insoluble fibre on Ca, Mg, Fe, Zn, Mn and Cu apparent absorption in rats. Arch Tierernahr 49, 293–299. 8988315 10.1080/17450399609381892

